# The Impact of a Heated Effleurage and Heated Tapotement Massage on Low-Back Discomfort from a Seat

**DOI:** 10.3390/bioengineering13060708

**Published:** 2026-06-20

**Authors:** Matt M. Mallette, Nathaniel Gur-Arie, Malak Almonjed, Nicola Gerrett

**Affiliations:** Integrative Human Research Lab, Gentherm, 38455 Hills Tech Dr., Farmington Hills, MI 48331, USA; matt.mallette@gentherm.com (M.M.M.); nathaniel.gur-arie@gentherm.com (N.G.-A.); malak.almonjed@gentherm.com (M.A.)

**Keywords:** low back pain, heated massage, myotonometry, pain perception, non-pharmacological, intervention

## Abstract

Lower back pain (LBP) is highly prevalent, and while non-pharmacological treatments exist such as heat or massage, they are rarely combined in a convenient manner. We examined the impact of two different heated massage protocols delivered from an automotive seat on LBP within typical commute times. Seventeen adults (eight females) with chronic, non-specific LBP (~6/10 initial back pain) evaluated a heated effleurage or heated tapotement massage—in a randomized order—applied to the lower back, upper thighs, and gluteal region while seated. Each visit included a 20 min control followed by a 20 min heated massage (30 min rest between), with thermal and subjective measurements assessed throughout. Before and after each 20 min session, viscoelastic properties of participants’ lower back muscles were assessed with a myotonometer. Heated massage increased skin temperature, thermal sensation and comfort vs. control (*p* ≤ 0.039). Both heated massage conditions reduced LBP at 10- and 20 min and reduced subjective tightness at 20 min (*p* ≤ 0.023). Tapotement produced an earlier reduction in tightness at 10 min and had a greater reduction than effleurage at 20 min (*p* ≤ 0.026). Increased tissue elasticity was observed in the heated tapotement condition (*p* ≤ 0.031). Seat-based heated massage offers a convenient method to alleviate LBP, potentially from changes to posterior chain tissue properties.

## 1. Introduction

Non-specific lower back pain (LBP) is one of the most common musculoskeletal complaints world-wide, with the Global Burden of Disease Study indicating over half a billion people suffering from it at any given time, and the number of cases is estimated to increase to 843 million by 2050 [1]. It is experienced by almost all adults, with ~80% reporting LBP at some point in their lives [2,3]. Non-specific back pain is the most common type of LBP reported and is defined as when the pathoanatomical cause of pain is unknown [3], but can originate from physical factors such as repetitive lifting, unhealthy lifestyles (e.g., obesity or sedentary), tight posterior chain muscles, or it can develop over time with aging [2]. Treatment for LBP spans several domains including pharmacological, non-pharmaceutical, stretching, thermal therapy, and manual therapy interventions with varying degrees of benefit, effectiveness, and adherence [3,4,5,6].

While pharmaceutical options may be an easy and effective strategy for acutely reducing pain, there is a growing interest in non-pharmacological strategies as first-line treatment and to avoid unwanted side effects [7,8]. Several over-the-counter pharmaceutical options average less than a 1-unit reduction on a 0-to-10 pain scale for patients experiencing chronic LBP [9,10]. In general, a reduction in LBP of <1-unit on a 10-point scale is considered a small effect, a 1-to-2-unit reduction a moderate effect, and a >2-unit reduction a large effect [11].

Evidence supports the use of massage for acutely reducing chronic LBP severity; however, the broad variability in massage techniques, populations, durations, and pressures—in addition to the difficulty of conducting blind trials—contributes to inconsistent findings across meta-analyses and systematic reviews [3,8,12,13]. Indeed, these limitations may be minimized when mechanical massaging devices are used, such as a massage chairs or beds [14,15]. It has been suggested that effleurage massages stimulate the parasympathetic nervous system and enhance venous return, whereas tapotement massages stimulate tissues [16]. A popular new form of tapotement massage using handheld percussive massage guns demonstrates that 10–15 min of percussion massage two-to-three times per week for six weeks has been shown to reduce back pain [17,18].

Various massage patterns delivered from an automotive seat for one hour demonstrates increases in local skin temperature and tissue oxygenation, suggesting increased blood flow to the area [19]. For acute pain relief, massage stimulates large diameter Aβ nerve fibres, inhibiting the local pain signal via the gate-control theory [16,20]. We have previously demonstrated that a single 33 min exposure to a heated automotive seat can provide a ~1-unit reduction in pain in people experiencing acute, subacute, and chronic LBP [21]. The reduction in pain was likely due to local effects such as increased blood flow to the area from the release of local bioactive substances, such as histamine, prostaglandins, and bradykinin, as well as producing thermal analgesia from stimulation of local warm-sensitive thermoreceptors, as the skin temperature reached was too low to activate Transient Receptor Potential Vanilloid-1 (TRPV-1) receptors [22,23,24,25].

An objective biomechanical assessment on the musculature would help provide insights into the reported subjective improvements, often plaguing massage interventions. Myotonometry is a non-invasive method to gain these properties from the muscles and underlying tissues via the deformation of superficial tissues from the application of a non-therapeutic small force. Myotonometry has been shown to be sensitive to acute changes, such as before and after receiving two minutes of percussive massage to the hamstring [26]. A meta-analysis and individual studies consistently show that adults with chronic low-back pain exhibit increased muscle stiffness and tone in the erector spinae and multifidus compared with age-matched healthy controls [27,28].

As both local heating and massage act on similar pathways to reduce pain (e.g., gate control theory, increase local blood flow), as well as independent pathways (e.g., stimulation of different nerve fibres, mechanical deformation of the tissues), combining these two techniques may result in a synergistic benefit than either therapy alone. Percussion massage has demonstrated beneficial outcomes attributed to enhanced proprioceptive function, improved mechanoreceptor sensitivity, and modulation of pain, collectively contributing to better motor control, reduced discomfort, and increased confidence in movement [17,18,26,29]. Comfort technology in vehicles, such as heating, cooling, and massaging seats have been in the market for decades. While initially used to enhance comfort, there has been a shift in consumer mindset to enhance ergonomics and provide health and wellness support [30]. Indeed, there are reports of people using the heated seats to alleviate pain [31], as well as evidence supporting this claim [21]. With almost 90% of adults in the United States owning a vehicle and average work commute times increasing to ~26 min [32,33], there are increased opportunities to provide wellness features to vehicle occupants in a convenient manner on people’s daily commute.

This study aims to determine the potential pain-relieving aspects of a heated effleurage massage compared to a heated tapotement massage delivered from an automotive seat on participants experiencing chronic non-specific LBP. We have previously shown that ~30 min of low-back heating can alleviate LBP [21], and this study investigates the addition of heating and massage on LBP in a shorter time frame. We hypothesize that both heated massages will reduce subjective back pain, but that the tapotement massage will have a larger impact on tissue properties.

## 2. Materials and Methods

### 2.1. Informed Consent

This study was approved by an Institutional Review Board (Protocol code: 11891, SterlingIRB, Atlanta, GA, USA) and conformed to all standards within the Declaration of Helsinki. All participants were informed of the experimental protocol and risks and provided verbal and written informed consent.

### 2.2. Participants

Twenty participants were recruited via an external recruitment agency (Cypher Research, Livonia, MI, USA); however, three participants were excluded from the analysis for not experiencing pain during the baseline on one of the two testing days upon arrival, as they did not represent the target population of individuals with back pain. Seventeen adults completed both test sessions, with nine males and eight females (age 46 ± 11 years, height 171 ± 11 cm, weight 77 ± 17 kg, body mass index 25.8 ± 2.8). Upon arrival, their heart rate was 68 ± 11 bpm, their systolic blood pressure was 120 ± 14 mm Hg, and their diastolic blood pressure was 77 ± 10 mm Hg, resulting in a mean arterial pressure of 91 ± 11 mm Hg. Detailed demographic information is in Table 1. Sample size estimates were calculated a priori using data reported in studies examining heat from an automotive seat [21] and a multi-dimensional massage protocol (e.g., effleurage, petrissage, and acupressure) [34]. Power analyses based on within-subject reductions in pain from these studies indicated required sample sizes of 15 and three people, respectively. Because the current protocol combines both modalities, a sample of 20 participants was chosen to exceed the larger estimate and ensure adequate statistical power.

Participants were included if they were currently experiencing non-specific lower back pain for longer than 3 months with a subjective pain of at least 3 out of 10 (with 0 equal to no pain and 10 equal to the worst pain imaginable) 5 days of the week. To ensure participants had real-world impairment to daily activities, inclusion required a score of >5 on the Physical Function subscale of the 36-Item Short Form Health Survey (SF-36) [35]. This subscale includes ten items scored as 2 (‘Yes, limited a lot’), 1 (‘Yes, limited a little’), or 0 (‘No, not limited at all’), with higher scores indicating greater functional limitation. Participants were excluded if they had specific LBP or had any other underlying health conditions, such as sensory impairments, type I or II diabetes, radiculopathy, or a body mass index classed as obesity or greater (≥30).

The SF-36 is a validated health-related quality of life questionnaire across eight domains of physical and mental health [35]. The physical functioning sub-component was used as an inclusion criteria because it focuses on everyday activities—such as walking, lifting, and climbing stairs—and is sensitive to musculoskeletal conditions such as back pain [35]. The SF-36 data are presented in Table 1 and scored as described [36].

### 2.3. Experimental Design

In a repeated-measures design, participants came to the laboratory on two occasions to have a control condition followed by either a heated effleurage or tapotement massage, in a cross-over design (see Table 2 for an overview of the test schedule). While each test day began with a 20 min baseline control, the heated effleurage or tapotement massage condition was assigned in a random order. The baseline procedures, heating protocol, and massage location were replicated exactly on each test day; the only difference was the 8 Hz pulsation of the air cells in the tapotement session. Each day, the control condition occurred first: participants sat in a car seat for 20 min with no thermal or pneumatic effectors. Thirty minutes later, one of the two 20 min massage programs began. The effleurage massage condition involved 20 min of applying force to one side of the lower back and contralateral upper thigh for ~4 s, then deflating and applying pressure to the opposite sides of the lower back and thigh. The tapotement massage used the same pattern; however, it was overlayed with a pulsating frequency of 8 Hz. During both massage conditions, the thermal technology in the lower third of the seat back and seat cushion was increased to a surface temperature of ~42.5 °C and ~37.5 °C, respectively. Before and after each control test and massage test, muscle and tissue viscoelastic and biomechanical properties were assessed with a MyotonPro (Myoton AS, Tallinn, Estonia), followed by three sit-and-reach tests separated by ~15 s.

### 2.4. Study Protocol

Following a telephone screening by the recruitment agency, eligible participants were invited to the laboratory for two days at the same time of day, separated by at least 48 h. On the first day, inclusion and exclusion criteria were confirmed, then the participants were given an overview of the project, and verbal and written informed consent was provided. After consent was provided, the participants completed a back pain information form, SF-36, and the Roland Morris Disability Questionnaire (RMDQ) [37]. The RMDQ was completed each test day and is a validated 24-item questionnaire designed to measure physical disability specific to low-back pain [37]. Then participants changed into a T-shirt and shorts to ensure consistent clothing insulation.

Participants then lay prone on a massage table and had their left and right erector spinae longissimus and multifidus muscles identified via manual palpation and marked according to SENIAM guidelines [38]. The erector spinae muscles were located by manually palpating the 12th rib and tracing it to the spine to locate the T12 vertebrae. L1 was then located, and the erector spinae were identified ~2 cm from either side of the spinous process. Each multifidus muscle was located by manually palpating the top of the iliac crest, moving to the spine at L4, and then palpating to L5; the posterior superior iliac spine was also located by manual palpation. Then, a line was drawn connecting the posterior superior iliac spine and L1, and the multifidus was identified along that line at the level of L5. Biomechanical and viscoelastic properties of the identified muscles were assessed with a handheld digital palpation device (MyotonPro). As the MyotonPro has been reported to provide reliable measurements to a depth of ~2 cm [39,40], the recorded values reflect the combined properties of the superficial lumbar extensor myofascial and surrounding tissues rather than the deep multifidus and erector spinae muscle in isolation. Following the assessment of muscle properties, three sit-and-reach tests were performed with ~15 s between each attempt.

Participants then stood next to the test seat and had four T-type thermocouples (Temprel, Boyne City, OH, USA) affixed to their back and two thermocouples affixed to their upper thigh using tape (Cover Roll Stretch, BSN Medical GmbH, Germany). For the back, a thermocouple was affixed on the right and left side of the middle and lower back, and for the thighs, a thermocouple was taped to each upper thigh. The environmental temperature was maintained at 22.5 ± 0.9 °C throughout the experiment to reflect steady-state temperatures inside a vehicle cabin. Participants then sat in the seat and were instructed to record their subjective thermal and pain-related sensations on a custom application (Labview, v.2021 SP1, National Instruments, Austin, TX, USA), which were logged every 10 s. After 20 min, the thermocouples were removed, and the muscle property assessment and sit-and-reach were performed. Participants then rested for 30 min in a position that did not elicit pain; then, they completed the same sequence as above (pre-test measurements, sitting in car seat, post-test measurements); however, this time they had either the effleurage or tapotement heated massage conditions for 20 min. After these post-massage measures, testing was concluded. Participants returned for the second and final day of testing 48 h later, which was identical to the first day of testing but had the other massage condition performed.

### 2.5. Outcomes and Measures

Participants were freely able to make changes whenever they perceived a change to their thermal, pain, or tightness sensations. Participants were asked to rate the thermal comfort and thermal sensation of their back and buttocks based on the ISO 10551 scales [41,42] throughout the test, as well as subjective back pain and subjective back tightness, which were all measured on a 0-to-10 scale. For back pain, the anchors were “no pain” and “worst pain imaginable” and for tightness, the anchors were “no tightness” and “worst tightness imaginable”.

The MyotonPro measures oscillation frequency (Hz), which provides an indication of intrinsic muscle tension (muscle tone), stiffness (N/m), which is a measure of the resistance to external force, and logarithmic decrement which is the muscle’s ability to disperse energy in response to a mechanical deformation [43]. Due to the inverse relationship, a lower decrement value represents increased elasticity. Following a perpendicular preload of 0.18 N, three impulses of 0.4 N lasting 15 ms were applied to the tissue. This device records the physical displacement and oscillation acceleration to calculate muscle tone, stiffness, and elasticity. When lying prone to assess muscle stiffness of the erector spinae, the MyotonPro has been demonstrated to have excellent intra-rater (intraclass correlation coefficient (ICC) = 0.88–0.91) and good inter-rater (ICC = 0.84–0.87) reliability, with a typical error measurement (SEM) of 24.6 N/m and 31.27 N/m, respectively [44]. Additionally, the MyotonPro has demonstrated excellent inter-rater reliability in an elderly population with chronic LBP in muscle tone (ICC = 0.92–0.94; SEM 0.53–0.59), stiffness (ICC = 0.94–0.95; SEM 17.03–18.02), and decrement (ICC = 0.90–0.94; SEM 0.09–0.04), supporting its use as a reliable and objective tool for assessing biomechanical muscle properties [28]. In healthy controls, the MyotonPro has demonstrated excellent within-day reliability for tone (ICC = 0.91–0.99; SEM 0.34–0.44), stiffness (ICC = 0.92–0.99; SEM 13.24–16.38), and good-to-excellent reliability for decrement (ICC = 0.81–0.98; SEM 0.09–0.17) [40]. Similarly, it demonstrated between-day reliability for both tone and stiffness (ICC ≥ 0.91); however, the between-day reliability for decrement was lower (ICC = 0.56–0.94; SEM 0.09–0.14) [40]. To ensure consistency, a coefficient of variation of <3% between all three measurements was required before proceeding to the next muscle.

Range of motion was assessed with a sit-and-reach test. Participants sat on a yoga-mat and, with their shoes removed, placed their feet on a vertical support with a ruler affixed to the top. With straight legs, participants were asked to reach forward with their arms extended as far as they could without exacerbating pain and hold that for one-second before relaxing. Participants then rested for 15 s before repeating the process three times in total. The sit-and-reach test was performed immediately after the myotonometry assessment before and after each control and massage condition.

### 2.6. Data and Statistical Analysis

Data were time-synced, formatted, visualized, and analyzed using Python (v3.12.0). The thermocouples were averaged by “level” such that there was a single mid-back, single low-back, and single thigh measurement. Missing temperature data was replaced within the “equivalent level” of the body segment (e.g., if the left-mid-back was missing entirely, it was replaced with the data for the right-mid-back, within the same participant and condition). For all continuous measures the mean of the first, tenth, and last minutes of data were calculated to evaluate differences.

All statistical measures were performed in JASP [45], with statistical significance set at *p* < 0.05. Data presented in-text are mean ± standard deviation (SD), whereas figures are mean ± standard error of the mean (SEM), unless otherwise reported. Effect sizes for pairwise comparisons were reported as Cohen’s *d* and interpreted as trivial (<0.2), small (0.2–0.5), moderate (0.5–0.8), and large (>0.8) [46].

For repeated-measures ANOVA models, normality was assessed by visual inspection of residual Q-Q plots. Sphericity was assessed using Mauchly’s test, and Greenhouse–Geisser corrections were applied when sphericity was violated. Significant effects were explored using Holm-adjusted pairwise post hoc comparisons.

Subjective pain, subjective tightness, temperature, thermal comfort, and thermal sensation were analyzed using repeated-measures ANOVA with condition and time as within-subject factors. Muscle mechanical properties were analyzed separately for each muscle and side, with pre-to-post-change scores for frequency, stiffness, and decrement compared across conditions using repeated-measures ANOVA. Range of motion was similarly analyzed using pre-to-post-change scores compared across conditions. Because multiple secondary and exploratory outcomes were evaluated, findings beyond the primary pain outcome were interpreted cautiously.

## 3. Results

### 3.1. Temperature Measurement and Perception Validation

**Figure 1:**
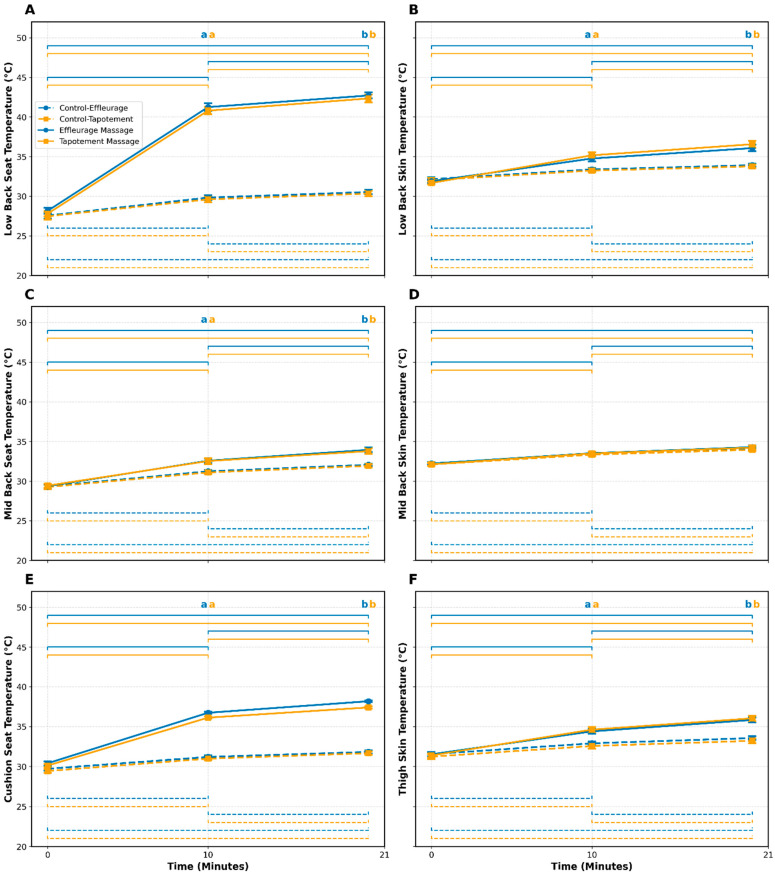
Seat surface temperatures of the lower back (**A**), mid-back (**C**), and cushion (**E**), and corresponding skin temperature of the lower back (**B**), middle back (**D**), and thighs (**F**). Data are presented as mean ± standard error of the mean (SEM). The effleurage massage (solid line) and control-effleurage (dashed line) are in blue and the tapotement massage (solid line) and control-tapotement (dashed line) are in yellow. The solid and dashed statistical markers indicated within-condition differences from 0 to 10 min, 0 to 20 min, and 10 to 20 min and are color coded to indicated which condition they refer to. The between-condition differences are color coded and indicate that massage is different from the corresponding control condition at 10 min (a) and 20 min (b).

**Figure 2:**
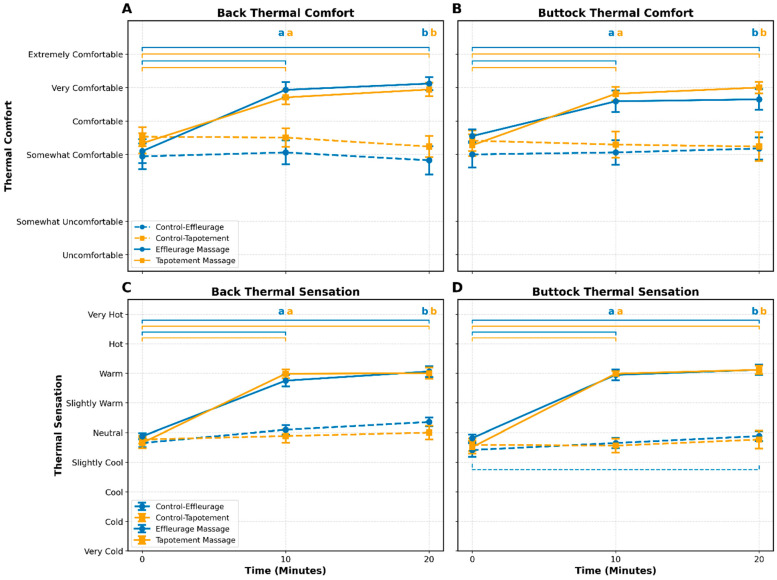
Thermal comfort and thermal sensation of the back (thermal comfort, (**A**); thermal sensation (**C**)) and the buttocks (thermal comfort, (**B**); thermal sensation, (**D**)). The effleurage massage (solid line) and control-effleurage (dashed line) are in blue and the tapotement massage (solid line) and control-tapotement (dashed line) are in yellow. Data are presented as mean ± SEM. The solid and dashed statistical markers indicated within-condition differences from 0 to 10 min, 0 to 20 min, and 10 to 20 min and are color coded to indicated which condition they refer to. The between condition-differences are color coded and indicate that massage is different from the corresponding control condition at 10 min (a) and 20 min (b).

Temperature (Figure 1) and thermal perception measures (Figure 2) confirmed that the heating intervention worked as intended. Relative to control, both heated massage conditions increased seat surface temperature of the lower back, middle back, and seat cushion (Figure 1A,C,E), with corresponding increases in skin temperature at the lower back and thighs (Figure 1B,F), as well as increased back and buttocks thermal comfort (Figure 2A,B) and thermal sensation (Figure 2C,D) (all d ≥ 1.10, *p* ≤ 0.039). These effects were evident by 10 min and maintained at 20 min. Mid-back skin temperature did not differ between conditions because no active heating was applied to that seat region (Figure 1D, d ≤ 0.33, *p* ≥ 0.109).

Within-condition changes over time were consistent with these between-condition findings, with greater increases in thermal comfort and sensation observed in the heated massage conditions than in the control conditions. No consistent differences were observed between massage types, supporting comparable thermal delivery across interventions.

### 3.2. Subjective Pain and Tightness Ratings

**Figure 3:**
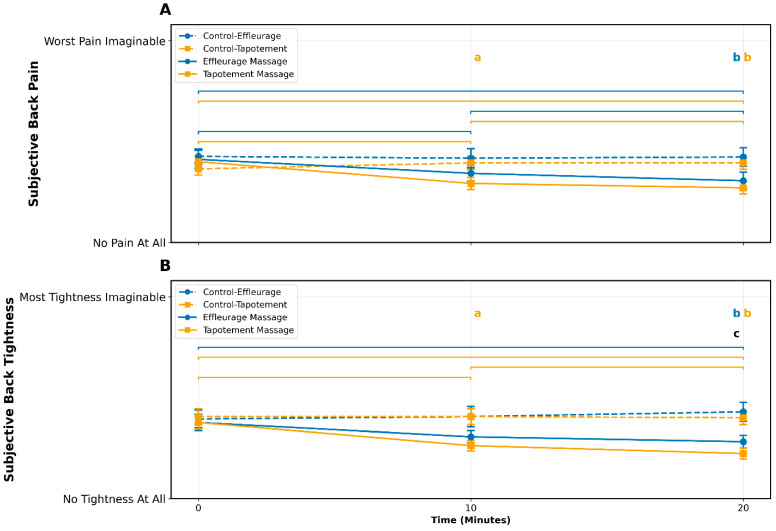
Subjective back pain (**A**) and subjective back tightness (**B**). The effleurage massage (solid line) and control-effleurage (dashed line) are in blue and the tapotement massage (solid line) and control-tapotement (dashed line) are in yellow. Data are presented as mean ± SEM. The solid and dashed statistical markers indicated within-condition differences from 0 to 10 min, 0 to 20 min, and 10 to 20 min and are color coded to indicated which condition they refer to. The between-condition differences are color coded and indicate that massage is different from the corresponding control condition at 10 min (a) and 20 min (b). Between-massage differences at 20 min are indicated by (c).

Subjective back pain (Figure 3A) and subjective back tightness (Figure 3B) were analyzed using RM ANOVA with Greenhouse–Geisser correction due to violation of sphericity. For subjective pain, significant main effects were observed for condition, *F*(1.670, 26.724) = 4.548, *p* = 0.025, time *F*(1.186, 18.978) = 10.617, *p* = 0.003, with a significant condition × time interaction also being observed: *F*(1.846, 29.543) = 6.835, *p* = 0.004. For subjective tightness, significant main effects were also observed for condition, *F*(1.963, 31.416) = 8.435, *p* = 0.001, and time, *F*(1.215, 19.445) = 11.437, *p* = 0.002, with a significant condition × time interaction, *F*(2.309, 36.946) = 8.949, *p* < 0.001.

Holm-adjusted post hoc comparisons showed no baseline differences among the prespecified condition contrasts for either pain or tightness. The two control conditions did not differ at any time point for subjective pain (d ≤ 0.40, *p* ≥ 0.463) or tightness (d ≤ 0.19, *p* ≥ 0.351). Relative to its matched control, tapotement massage decreased subjective pain and tightness at 10 min (pain, d = 0.66, *p* = 0.007; tightness, d = 0.93, *p* = 0.006) and 20 min (pain, d = 0.81, *p* = 0.005; tightness, d = 1.15, *p* < 0.001). Compared to its matched control, effleurage massage did not decrease pain or tightness at 10 min (pain, d = 0.49, *p* = 0.090; tightness, d = 0.65, *p* = 0.116) but did at 20 min (pain d = 0.77, *p* = 0.014; tightness d = 0.96, *p* = 0.009). The two massage conditions did not differ in pain at any time point (d ≤ 0.33, *p* ≥ 0.260); however, tightness was lower in the tapotement massage compared to the effleurage at 20 min (d = 0.38, *p* = 0.026).

Within conditions showed that neither control condition impacted subjective pain or tightness over time (pain, d ≤ 0.19, *p* ≥ 0.617; tightness, d ≤ 0.27, *p* ≥ 0.087). Both massage conditions reduced pain over time. In the effleurage condition, pain was lower at 10 min and 20 min relative to the baseline and was further reduced at 20 min relative to 10 min (all d ≥ 0.24, *p* ≤ 0.023). In the tapotement condition, pain was also lower at 10 min and 20 min relative to the baseline and lower again at 20 min relative to 10 min (all d ≥ 0.14, *p* ≤ 0.033). For tightness, effleurage showed a reduction at 20 min relative to the baseline (d = 0.61, *p* = 0.032), but not at 10 min (d = 0.46, *p* = 0.080). In contrast, tapotement reduced tightness at both 10 min and 20 min relative to the baseline (both d ≥ 0.74, *p* ≤ 0.003), with a further reduction from 10 min to 20 min (d = 0.26, *p* = 0.016).

### 3.3. Muscle Properties

**Figure 4:**
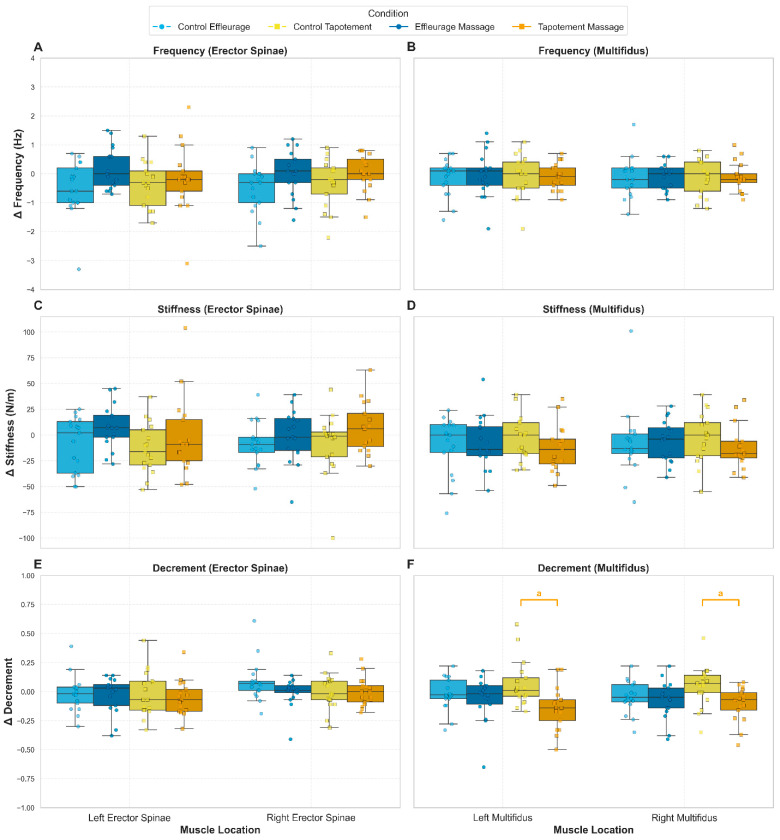
Muscle property differences in the erector spinae (frequency, (**A**); stiffness, (**C**); decrement (**E**)) and multifidus (frequency, (**B**); stiffness, (**D**); decrement (**F**)) from before and after each condition (control-effleurage, light blue; effleurage massage, dark blue; control-tapotement, yellow; tapotement massage, orange). The left set of data on each graph is data from the left side of the body, and the right set of data is from the right side of the body. Data are displayed as box-and-whisker plots with the median indicated within each box, and the upper and lower bounds are made of up of the 75th and 25th percentiles, respectively. The whiskers extend to the minimum and maximum data, excluding outliers. Individual participants are displayed as color-coded squares for each condition. Differences from massage to its matched control are indicated by (a).

RM ANOVAs were conducted on pre-to-post-change scores for each condition. For muscle tone (Figure 4A,B, frequency, Hz) and muscle stiffness (Figure 4C,D, Nm), no significant condition effects were observed in either the erector spinae or multifidus on either side (muscle tone *p* ≥ 0.111; muscle stiffness *p* ≥ 0.216). For decrement (Figure 4E,F), no significant condition effects were detected in either the right or left erector spinae (both *p* ≥ 0.208). However, decrement differed significantly by condition in both the right and left multifidus (both *p* ≤ 0.031). Holm-adjusted post hoc comparisons showed greater reductions in decrement during tapotement massage to its matched control for both the right (d = 0.98, *p* = 0.031) and left (d = 1.16, *p* = 0.007) multifidus.

### 3.4. Range of Motion

Range of motion change scores did not differ significantly across conditions, *F*(3, 48) = 0.993, *p* = 0.404. Mean pre-to-post-changes were 0.5 ± 1.4 cm for the effleurage control, 1.0 ± 2.1 cm for the tapotement-control, 1.5 ± 1.9 cm for the effleurage massage, and 1.4 ± 2.1 cm for the tapotement massage conditions. Although the massage conditions demonstrated numerically greater improvements, Holm-adjusted post hoc comparisons showed no significant differences between any conditions (all d ≤ 0.54, *p* ≥ 0.809).

## 4. Discussion

We examined the impact of two different 20 min heated massages on subjective back pain, subjective back tightness, and muscle properties, delivered from an automotive seat. We found that both a heated effleurage and tapotement massage delivered to the lower back, gluteal, and upper thigh regions similarly reduced subjective back pain. The tapotement massage provided a greater benefit to subcutaneous tissue elasticity properties and had a faster and greater perceived impact alleviating subjective back tightness. This intervention is designed to reduce pain in a way to easily fit into the driver’s (or passenger’s) daily life without having to take any additional actions to achieve pain relief. Practitioners often see poor retention rates with traditional at-home therapies which involve dedicating periods of time several times per week to achieve any pain relief [47]. The pain-relieving methods here are designed to easily fit into a person’s daily routine and do not require any additional effort to get relief. We intentionally chose a short intervention time (20 min) as this is within the average drive times in North America, Europe, and Asia [33,48,49].

During each 20 min heated massage intervention, participants experienced a decrease in subjective back pain by ~1 unit, a moderate effect [11]; whereas sitting in a seat with no thermal or massaging effectors for 20 min had no impact on subjective pain or tightness. We previously showed a benefit from 33 min of localized heating delivered to the lower back having a 1-unit reduction on pain [21]. Likewise to our previous study, seat temperature of the lower third of the seat back increased to ~40 °C after 5 min, and increased to ~42.5 °C throughout the remaining 15 min. Observed skin temperature in this study is lower than our previous study from a similar seat surface temperature profile; however, upon inspection of individual data, we noticed that the placement of the lower back thermocouples were placed too superior—approaching the mid-back—on some participants. When those participants were removed, lower back skin temperature reached ~38 °C compared to ~36 °C with all participants. Changes in skin temperature likely reflect both exogenous heating and physiological responses from mechanical stimulation. Similar to our previous study [21], the skin temperatures reached would have been too low to activate TRPV-1 receptors to inhibit pain receptors [24,25]; therefore, other mechanisms might have contributed to reduce the subjective pain experienced, such as increased blood flow, increased local metabolism, and activation of warm-sensitive thermoreceptors to block pain signals and withdrawal sympathetic tone [20,23,50]. In addition to thermally mediated mechanisms, the mechanical stimulation delivered through the seat may have independently contributed to pain relief through activation of cutaneous mechanoreceptors and spinal gate control—a mechanism particularly relevant to the tapotement condition employed in this study [22,51]. These proposed mechanisms are based on the previous literature and should be considered potential explanations only, as the present study did not directly measure the physiological processes involved.

A novel feature of this study was implementing a tapotement-style massage into an automotive seat, providing a mechanical analgesic stimulus that may have contributed alongside the concurrent thermal intervention to reduce subjective pain. Stimulation of cutaneous mechanoreceptors generates large-diameter Aβ afferent input that, via inhibitory interneurons, suppresses ascending nociceptive signals—a spinal gating mechanism described by gate control theory [22,52,53,54]. For example, when applying vibration (20 to 230 Hz) near painful radiant heat attenuates nociception, indicating that mechanoreceptive channels contribute to pain reduction via spinal inhibition of ascending nociceptive input [55]. The sustained pressure applied through the seat interface would likely activate slowly adapting mechanoreceptors—specifically Merkel discs (SA1), which encode ongoing pressure magnitude, and Ruffini endings (SA2), which respond to skin stretch and tissue deformation under load—both transmitting via large-diameter Aβ afferents to engage inhibitory interneurons in the dorsal horn [56]. Superimposing an 8 Hz pulsation onto this sustained pressure would, theoretically, additionally recruit rapidly adapting Meissner corpuscles (RA1), which are optimally tuned to low-frequency flutter in the 5–50 Hz range, broadening the mechanoreceptor complement and increasing the magnitude of the large-diameter Aβ afferent input to the dorsal horn beyond that achieved by sustained pressure alone [52]. Together, these receptors likely increase Aβ afferent input, which may contribute to spinal gating mechanisms described by the gate control theory [20,52], consistent with the reduction in subjective pain observed in this study. Furthermore, the combined dual heat and mechanical stimulation may have contributed to the observed analgesic effects, with thermally activated warm-sensitive afferents and mechanoreceptor-driven Aβ input converging on shared inhibitory circuits in the dorsal horn to produce reduced suppression of nociceptive afferents than either modality alone [20,51]. Although the present study did not directly quantify mechanoreceptor activation, Aβ afferent activity, or spinal inhibitory processes, the observed reductions in subjective pain are consistent with mechanisms proposed involved with the gate control theory and thermal analgesia, suggesting that neurophysiological pathways may have contributed to the analgesic effects observed here.

Most investigations with massage for pain reduction use a combination of massage styles, such as Swedish, hot stone, Thai, sports, etc., that often blend effleurage, tapotement, and percussive techniques. Because the device examined consists of pneumatic air cells, we were able to provide strictly effleurage or tapotement massages. Previous work carried out with handheld percussive massage devices (~46 Hz) have found increases in range of motion, resulting from increased pliability and muscle elasticity and decreased muscle stiffness [29]. While both massages similarly improved range of motion and reduced subjective LBP, the tapotement massage produced a larger pain-relieving effect compared to effleurage massage at both 10 min and 20 min. Further, the tapotement massage increased tissue elasticity (from decreased decrement) and had a larger and faster impact on subjective back tightness, implying that a heated tapotement massage at a slower frequency than previously published (~8 Hz) can have positive impacts on perceived pain and muscle-related outcomes. These decreases in decrement indicate a positive shift, as lower decrement values are similar to those with a “pain-free” back, compared to those with back pain using the same measurement device [28]. As muscular properties were not our primary variables, we are likely underpowered for these variables; however, the variability of these measures are within published norms [28,44]. A potential reason for observing larger changes in the multifidus may have been due to the zonal lower back heating, and in some individuals the erector spinae muscle group was above the level of the heat.

Although both massage conditions showed numerically greater increases in range of motion than the control conditions, these differences were not statistically significant across conditions and should therefore be interpreted cautiously. In contrast, decrement was reduced in the multifidus following tapotement massage relative to its matched control, suggesting a possible localized effect on muscle mechanical behavior. This may be relevant because altered posterior-chain flexibility and impaired trunk function are commonly associated with low-back pain and disability [57,58]. However, sit-and-reach performance is influenced by several factors beyond lumbar tissues, including hamstring flexibility, thoracic spine motion, and hip mobility, and the present data therefore do not support strong conclusions regarding functional flexibility improvements. In addition, although the study was powered for pain, it was likely underpowered for smaller effects in secondary biomechanical and functional outcomes. Larger studies using more region-specific functional assessments are needed to determine whether these mechanical changes translate into meaningful mobility benefits in individuals with chronic low-back pain.

### Experimental Limitations

A key limitation of this study is that several aspects of the protocol were intentionally designed to maximize ecological validity and real-world applicability. First, both heating and massage were combined into a single intervention, reflecting the way these features are typically implemented in production seats equipped with a multi-bladder pneumatic massage system. Because heat and massage were not examined in isolation, the relative contribution of each modality to the observed effects cannot be determined. However, this combined approach was selected deliberately to evaluate the intervention as it would be experienced by end users and to extend our prior work demonstrating beneficial effects of heating alone on subjective low-back pain [21]. Similarly, seat heating was intentionally maintained below the automotive guideline threshold of <44 °C [59] to reflect realistic and safe in-vehicle operation. This conservative thermal exposure may have limited the magnitude of pain-relieving effects, as skin temperature likely remained below the activation threshold of TRPV-1 receptors [25]. Nonetheless, no participants in the current study, or in our previous study using slightly higher seat surface temperatures, reported thermal discomfort, supporting the tolerability with this level of heating [21]. In addition, post-intervention assessments were conducted while skin temperature remained elevated, consistent with the aim of evaluating the acute combined effects of thermal massage as experienced in a real-world setting. This design allowed assessment of the immediate impact of the intervention, but it also prevented separation of the independent effects of heat and massage on tissue properties.

Additional limitations should be addressed in future work. The fixed within-session order, in which the control condition always preceded the intervention, introduces the possibility of order effects such as familiarization or time-dependent changes that cannot be fully excluded despite the 30 min rest period. Future studies would benefit from a randomized or counterbalanced condition order. Moreover, inclusion of heat-only, massage-only, and combined intervention arms would help clarify the mechanisms underlying pain reduction, while a recovery period before post-intervention assessment would allow differentiation between transient thermal effects and changes attributable to massage.

Interpretation of the biomechanical outcomes should also be approached with caution. Logarithmic decrement was the only biomechanical parameter to demonstrate a significant change. Although the MyotonPro has shown excellent within-day [40] and inter-rater [28] reliability for frequency, stiffness, and decrement, decrement has been reported to have lower between-day reliability [40], which may limit confidence in this finding. In addition, while the study was powered for the primary outcome of pain, the final sample of 17 participants was likely underpowered for secondary biomechanical and functional outcomes. Accordingly, the directional changes observed in some secondary measures should be interpreted cautiously. A larger, fully powered randomized controlled trial informed by the effect sizes reported here is therefore warranted. Finally, because only a single intervention session was examined, it remains unclear whether repeated use of heated massage during regular driving would produce greater or more sustained pain-relieving benefits.

## 5. Conclusions

We demonstrate that a 20 min heated effleurage or heated tapotement massage delivered to the lower back and seat cushion from a car seat can alleviate subjective back pain and tightness in individuals with chronic, non-specific, lower back pain. Additionally, we found that both the heated effleurage and tapotement massages were able to beneficially change muscle stiffness and elastic characteristics, with larger changes elicited from the tapotement massage. By showing efficacious treatment effects with a shortened treatment time of 20 min compared to our previous work of 33 min, this enhances the ability to be used beneficially during each drive, which may have implications for pain alleviation with repeated use.

## 6. Patents

U.S. and international patent applications: US1212227 [60], DE102023103100B4 [61], and DE102019113629B4 [62].

## Figures and Tables

**Table 1:** Participant demographics and baseline characteristics. When measurements were collected each day, the scores are denoted by what massage type the participant received that day. The eight sub-sections to the SF-36 range from 0 to 100, with a higher score indicating better health-related quality of life.

Characteristic	
Back pain duration	>3 months & ≤12 months: 3 >1 year ≤ 5 years: 8 >5 years: 6
Back pain frequency (pain experienced per week)	Everyday: 9 5 or 6 days/week: 6 3 or 4 days/week: 2
Back pain trend over 3 months	Constant: 15 Increasing: 2
Typical back pain experienced (mean ± SD) [min to max]	5.6 ± 1.5 [3 to 8]
Baseline back pain on test days (mean ± SD) [min to max]	Effleurage day: 4.6 ± 1.5 [2 to 7] Tapotement day: 4.4 ± 1.5 [1 to 7]
Back tightness on test days (mean ± SD) [min to max]	Effleurage day: 4.6 ± 1.8 [2 to 9] Tapotement day: 4.6 ± 1.5 [2 to 7]
Rolland Morris disability questionnaire (Mean ± SD)	Effleurage day: 7.4 ± 3.4 Tapotement day: 8.0 ± 3.8
Medications	None: 7 OTC pain medications: 10 Cannabis: 1
Ongoing therapy	None: 17
SF-36 Score (mean ± SD)	
Physical functioning	63.24 ± 22.43
Role limitations (physical)	41.18 ± 37.44
Role limitations (emotional)	64.71 ± 39.39
Energy/fatigue	55.59 ± 20.45
Emotional well-being	75.29 ± 19.14
Social functioning	67.65 ± 19.79
Pain	47.79 ± 16.39
General health	63.24 ± 16.86

**Table 2:** Overview of the protocol and measures for each testing day. The intervention was either a heated effleurage or heated tapotement massage, conducted in randomized order. For the control condition carried out on the same day as the effleurage massage, it will be referred to control-effleurage, and the control performed the same day as the tapotement massage, it will be referred to as control-tapotement.

Pre-Test	Control	Post-Test	Rest	Pre-Test	Intervention	Post-Test
Myotonometry Assessment Sit-and-Reach	Subjective Back Pain Subjective Back Tightness Temperature	Myotonometry Assessment Sit-and-Reach	30 min rest	Myotonometry Assessment Sit-and-Reach	Subjective Back Pain Subjective Back Tightness Temperature	Myotonometry Assessment Sit-and-Reach

## Data Availability

The data are not publicly due to proprietary reasons.

## Abbreviations

The following abbreviations are used in this manuscript:

LBP	lower back pain
RM ANOVA	repeated-measures analysis of variance
RMDQ	Rolland Morris Disability Questionnaire
SF-36	36-Item Short Form Health Survey
SEM	standard error of the mean
SD	standard deviation
TRPV	Transient Receptor Potential Vanilloid
